# Carbon–carbon bond cleavage for Cu-mediated aromatic trifluoromethylations and pentafluoroethylations

**DOI:** 10.3762/bjoc.11.286

**Published:** 2015-12-18

**Authors:** Tsuyuka Sugiishi, Hideki Amii, Kohsuke Aikawa, Koichi Mikami

**Affiliations:** 1 Division of Molecular Science, Faculty of Science and Technology, Gunma University, 1-5-1 Tenjin-cho, Kiryu, Gunma 376-8515, Japan; 2 Department of Applied Chemistry, Graduate School of Science and Engineering, Tokyo Institute of Technology, O-okayama, Meguro-ku, Tokyo 152-8552, Japan

**Keywords:** β-carbon elimination, carbon–carbon bond cleavage, decarboxylation, tetrahedral intermediate, trifluoroacetate, fluoral, trifluoromethylation

## Abstract

This short review highlights the copper-mediated fluoroalkylation using perfluoroalkylated carboxylic acid derivatives. Carbon–carbon bond cleavage of perfluoroalkylated carboxylic acid derivatives takes place in fluoroalkylation reactions at high temperature (150–200 °C) or under basic conditions to generate fluoroalkyl anion sources for the formation of fluoroalkylcopper species. The fluoroalkylation reactions, which proceed through decarboxylation or tetrahedral intermediates, are useful protocols for the synthesis of fluoroalkylated aromatics.

## Introduction

Organofluorine compounds attract attention because of their applicability in various fields, such as medicine, agrochemical and material science. It has been widely reported that nearly 15% of pharmaceuticals and 20% of agrochemicals on the market contain fluorine atoms, including several of the top drugs. Of particular interest are compounds containing the structural motif of a (trifluoromethyl)aryl group (Ar–CF_3_) [1–7]. The characteristic size, strong electron-withdrawing ability, and the high lipophilicity of the trifluoromethyl group are key properties of biologically active CF_3_-containing molecules [8]. Perfluoroalkylcopper compounds (C*_n_*F_2_*_n_*_+1_Cu), which are soft and relatively stable perfluoroalkyl organometallic reagents (C*_n_*F_2_*_n_*_+1_M) with high reactivity, act as prominent cross-coupling participants in aromatic perfluoroalkylation reactions [9–32]. In order to prepare C*_n_*F_2_*_n_*_+1_Cu species, several representative protocols have been reported. Among these protocols, each method has individual merit. Particularly, Ruppert–Prakash reagents (C*_n_*F_2_*_n_*_+1_SiR_3_) have been used as the source of perfluoroalkyl anions (C*_n_*F_2_*_n_*_+1_^−^) for the generation of C*_n_*F_2_*_n_*_+1_Cu. However, perfluoroalkylsilane sources are costly for large-scale operation. On the other hand, economical and useful perfluoroalkylated carboxylic acid derivatives, such as perfluoroalkylated carboxylates (C*_n_*F_2_*_n_*_+1_CO_2_Na or C*_n_*F_2_*_n_*_+1_CO_2_K), halodifluoroacetates (XCF_2_CO_2_R), perfluoroalkyl carboxylates (C*_n_*F_2_*_n_*_+1_CO_2_R), perfluoroalkyl ketones (C*_n_*F_2_*_n_*_+1_COR), and hemiaminals derived from fluoral (CF_3_C(OSiMe_3_)NR_2_), can generate C*_n_*F_2_*_n_*_+1_Cu via carbon–carbon bond cleavage. Herein we focus on Cu-mediated perfluoroalkylation reactions through which carbon dioxide, the esters, or the *N*-formylamines are eliminated from the perfluoroalkyl reagents.

## Review

### Decarboxylation of perfluoroalkylacetates

**Scheme 1:**
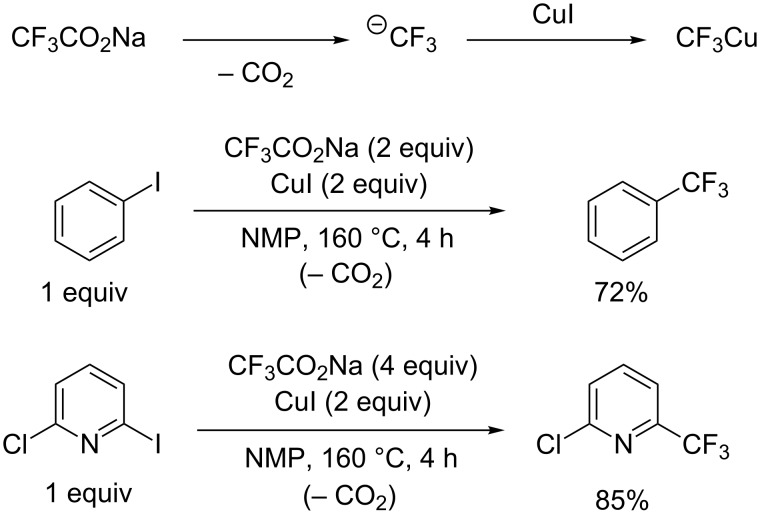
Trifluoromethylation using trifluoroacetate.

Trifluoroacetate salts are one of the most readily available trifluoromethylating agents compared to ozone-depleting CF_3_Br, and expensive CF_3_I. Sodium trifluoroacetate (CF_3_CO_2_Na) is a stable compound at room temperature. Under heating conditions (150–200 °C), CF_3_CO_2_Na plays the role of the CF_3_^−^ source and [CF_3_Cu] species with CuI are generated in situ. In the presence of CuI, CF_3_CO_2_Na undergoes trifluoromethylation with aryl halides via decarboxylation [33–34] (Scheme 1).

**Scheme 2:**
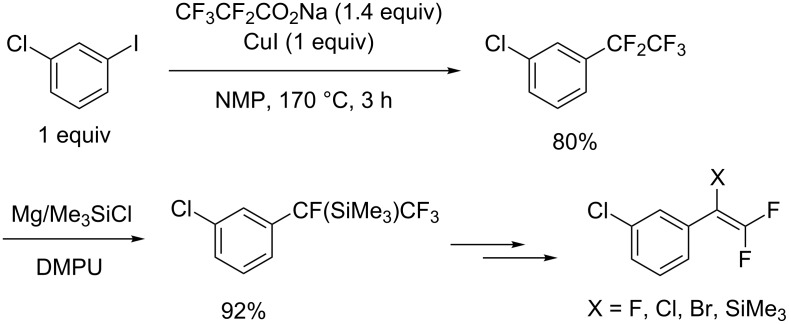
Decarboxylative pentafluoroethylation and its application.

A pentafluoroethyl group (C_2_F_5_) was fixed at the arene with sodium pentafluoropropionate [35] (Scheme 2). The reaction mechanism is similar to that of the trifluromethylation using CF_3_CO_2_Na [33–34]. Upon heating, the mixture of CF_3_CO_2_Na and CuI in NMP, 3-chloroiodobenzene underwent cross-coupling to provide the pentafluoroethylated compound in 80% yield. The pentafluoroethylated aromatic product was applied to the synthesis of 2,2-difluorostyrenes through Mg(0)-promoted defluorinative silylation followed by fluorine-ion-catalyzed 1,2-desilylative defluorination.

**Scheme 3:**
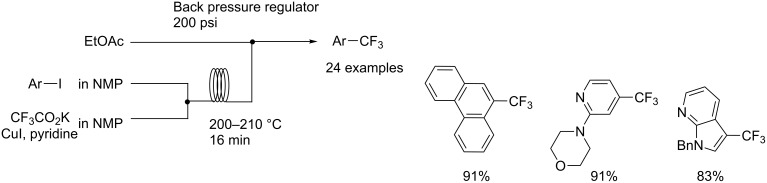
Trifluoromethyation with trifluoroacetate in a flow system.

Buchwald et al. demonstrated aromatic trifluoromethylation using potassium trifluoroacetate (CF_3_CO_2_K), CuI and pyridine under flow conditions. Increasing the reaction temperature from 160 °C to 200 °C accelerated the decarboxylation of CF_3_CO_2_K [36] (Scheme 3). The trifluoromethylation using a microreactor resulted in a good yield within a short reaction time by virtue of the thermal stability of CF_3_Cu and control of mixing. Taking advantage of the flow microreactor, a new protocol for scalable aromatic trifluoromethylation was developed.

**Scheme 4:**
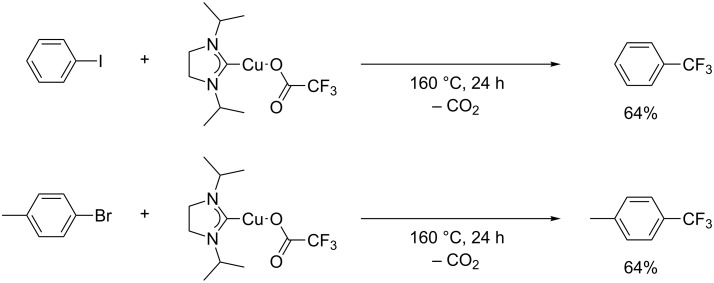
Trifluoromethylation of 4-bromotoluene by [(NHC)Cu(TFA)].

From a mechanistic aspect, Vicic and co-workers explored the direct generation of CF_3_Cu from CF_3_CO_2_Cu. The use of (N-heterocyclic carbene)copper-trifluoroacetates prepared from trifluoroacetic acid (TFA) was investigated in the decarboxylative trifluoromethylation of aryl halides [37] (Scheme 4). Not only iodobenzene but also 4-bromotoluene was trifluoromethylated by the [(NHC)Cu(TFA)] complex.

**Scheme 5:**
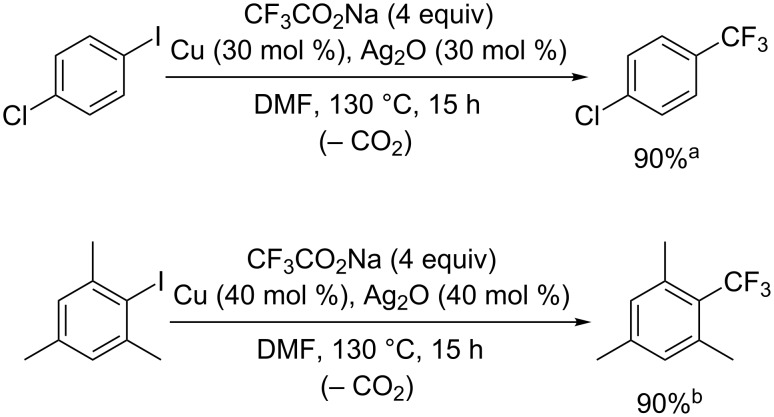
Trifluoromethylation of aryl iodides with small amounts of Cu and Ag_2_O. ^a^The yield was determined by GC analysis. ^b^The yield was determined by ^19^F NMR analysis using CF_3_CH_2_OH as an internal standard.

**Scheme 6:**
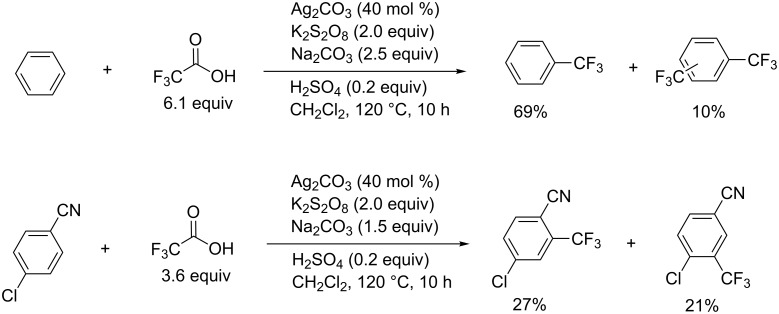
C–H trifluoromethylation of arenes using trifluoroacetic acid.

The perfluoroalkylation reactions mentioned above require a stoichiometric amount of copper reagent, whereas it was found that the addition of silver salts is effective for the copper-mediated trifluoromethylation of aryl iodides [38] (Scheme 5). The amount of copper used in the reaction was reduced to 30 or 40 mol % by adding a small amount of Ag_2_O. As a related decarboxylative transformation, silver-mediated aromatic trifluoromethylation was recently developed. Zhang et al. reported the direct aryl C–H trifluoromethylation in which TFA works as a trifluoromethylation reagent [39] (Scheme 6). In this reaction, TFA releases a CF_3_ radical via decarboxylation, which reacts with the arenes to yield trifluoromethyl-substituted products. This report suggests that TFA can act as a trifluoromethyl source in the reaction with inactivated aromatic compounds, while the control of regioselectivity is difficult.

### Trifluoromethylation with difluorocarbene and fluoride ions

**Scheme 7:**
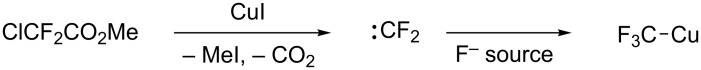
CF_3_Cu generated from chlorofluoroacetate and CuI.

The reaction system with ClCF_2_CO_2_Me/KF/CuI also generates CF_3_Cu in situ [40–41] (Scheme 7). The demethylation of ClCF_2_CO_2_Me proceeds by iodide, followed by decarboxylation of the resulting chlorodifluoroacetate to provide difluorocarbene (:CF_2_), trapped by fluoride to give the CF_3_^−^ species. This reacts with CuI leading to CF_3_Cu.

**Scheme 8:**
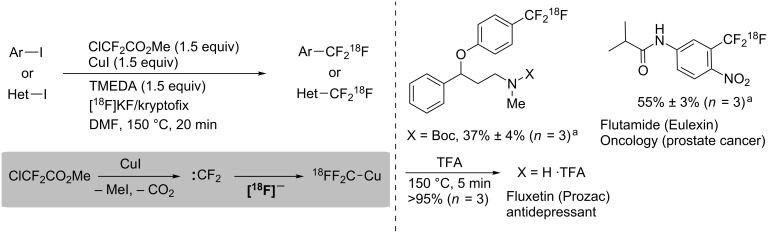
[^18^F]Trifluoromethyation with difluorocarbenes for PET. ^a^Radiochemical yield determined by HPLC.

The method described above for the trifluoromethylation of aryl iodides with ClCF_2_CO_2_Me and fluoride can be utilized for clinical studies. Herein, we introduce one example of decarboxylative [^18^F]trifluoromethylation for positron emission tomography (PET) studies. A synthetic methodology for [^18^F]labelled-CF_3_ arenes is desired for the application of PET imaging. The reason is that the [^18^F] isotope has a longer half-life (110 min) than ^13^N (10 min) or ^15^O (2 min); however, the incorporation of [^18^F] must be rapid and the use of the products containing [^18^F] must be immediate. Many of the reported strategies have a limited scope of starting materials or require expensive reagents and a multistep synthesis. The [^18^F]trifluoromethylation performed with commercially available reagents by using [^18^F]fluoride demands no complex such as [^18^F]CF_2_Cu, and thus the method should contribute to efficient PET imaging [42] (Scheme 8).

### Synthesis of perfluoroalkylcopper from perfluoroalkyl ketones or esters

**Scheme 9:**
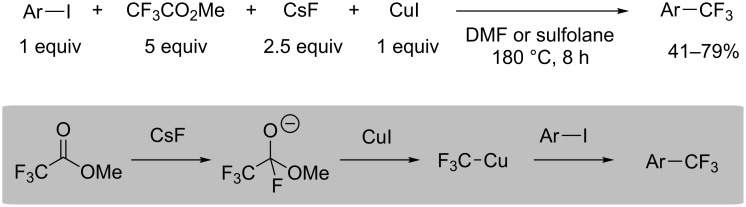
Trifluoromethylation with trifluoroacetate and copper iodide.

Langlois et al. reported that trifluoromethylation with methyl trifluoroacetate was successfully carried out in DMF or sulfolane at 180 °C [43] (Scheme 9). Methyl trifluoroacetate, which is more readily available than methyl chlorodifluoroacetate, acts as a trifluoromethylating agent. In this synthesis, the methyl trifluoroacetate/CsF/CuI system would form the tetrahedral intermediates to generate CF_3_Cu species in situ.

**Scheme 10:**
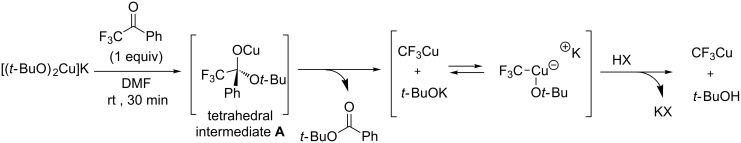
Preparation of trifluoromethylcopper from trifluoromethyl ketone.

**Scheme 11:**
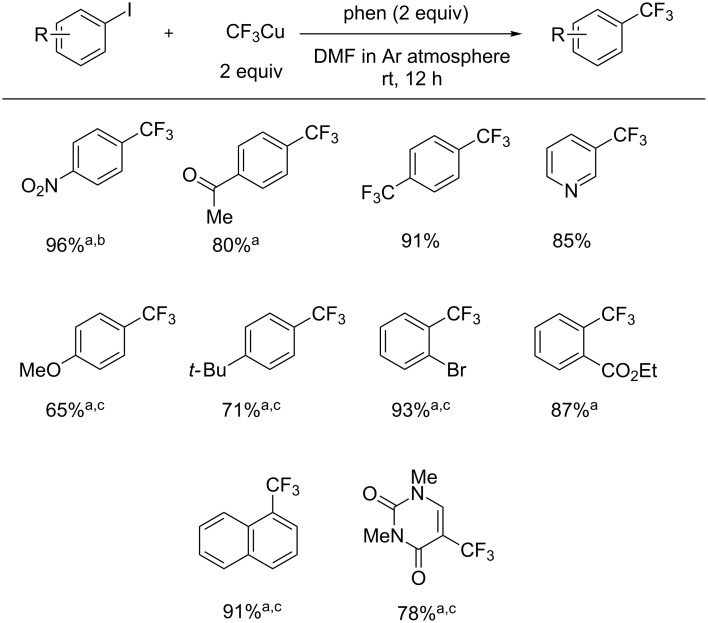
Trifluoromethylation of aryl iodides. ^a^Isolated yield. ^b^1 equivalent each of CF_3_Cu reagent and 1,10-phenanthroline were used. ^c^Reaction temperature was 50 °C.

Mikami and co-workers accomplished the synthesis of CF_3_Cu at room temperature with perfluoroalkyl ketone derivatives and appropriate nucleophiles. It is indicated that the CF_3_Cu reagent is directly formed from tetrahedral intermediate **A** [44] (Scheme 10). The CF_3_Cu reagent was applied to aromatic trifluoromethylation with aryl iodides, which have electron-withdrawing or electron-donating functional groups, in good to high yields (Scheme 11).

**Scheme 12:**
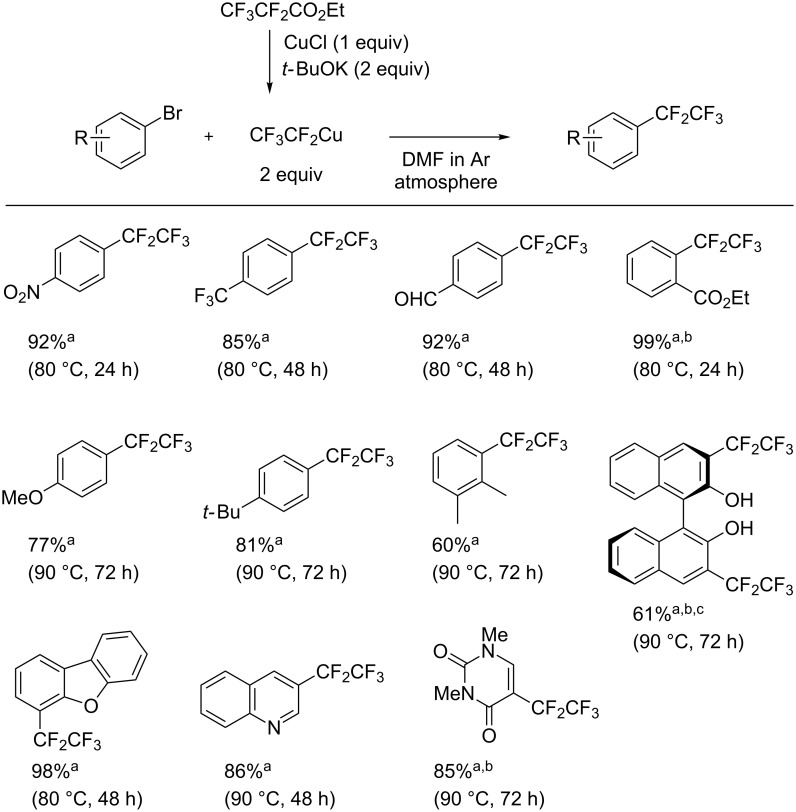
Pentafluoroethylation of aryl bromides. ^a^Yield was determined by ^19^F NMR analysis using benzotrifluoride (BTF) or (trifluoromethoxy)benzene as an internal standard. ^b^Isolated yield. ^c^4 equivalents of CF_3_CF_2_Cu reagent were used.

The preparation of the C_2_F_5_Cu reagent was investigated as well [45]. Pentafluoropropionate was reacted with CuCl salt in the presence of KO*t*-Bu to afford C_2_F_5_Cu. A variety of aryl bromides were reacted with C_2_F_5_Cu under the optimized conditions, providing pentafluoroethylated aryl products in moderate to high yield (Scheme 12).

**Scheme 13:**
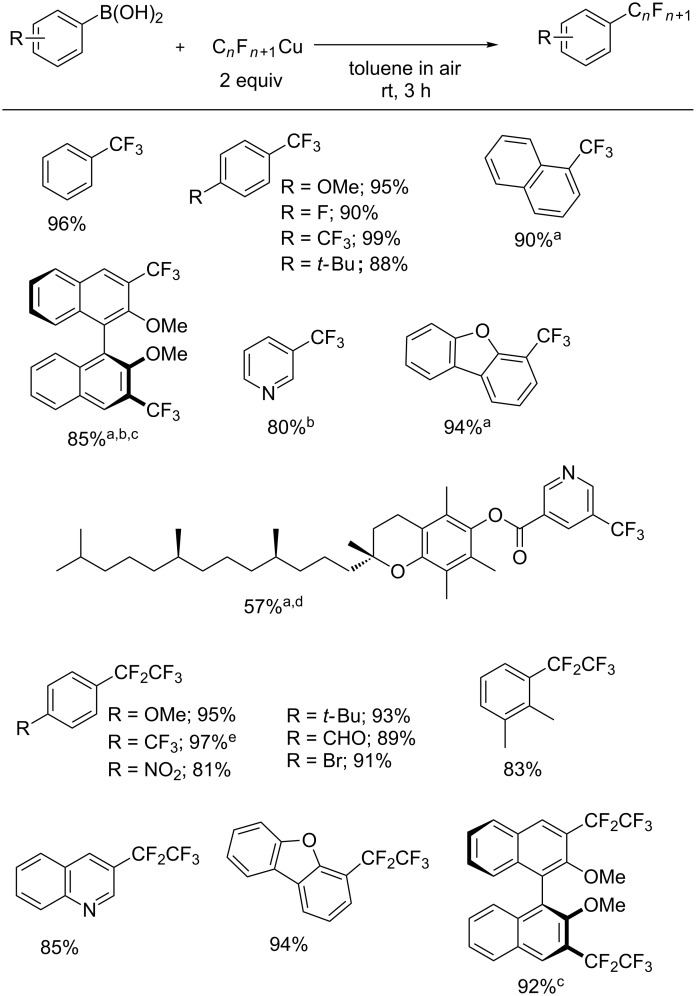
Perfluoroalkylation reactions of arylboronic acids. ^a^Isolated yield. ^b^DMF was used instead of toluene as a solvent. ^c^4 equivalents of C*_n_*F*_n_*_+1_Cu reagent were used. ^d^Pinacolboronate ester (Bpin) was used instead of boronic acid. ^e^Yield was determined by ^19^F NMR analysis using BTF as an internal standard.

The copper-mediated oxidative trifluoromethylation of arylboronic acids are important reactions in organic chemistry because arylboronic acids are widely used. Oxidative, aromatic perfluoroalkylation reactions with arylboronic acid derivatives have been studied by several groups. Qing et al. and Buchwald et al. used the Ruppert–Prakash reagent (CF_3_–SiMe_3_) directly as a CF_3_^−^ source [46–47]. From CF_3_–SiMe_3_, Hartwig et al. developed a new combination of Ir-catalyzed C–H borylation and oxidative cross-coupling using [(phen)CF_3_Cu] [48]. Grushin et al. utilized fluoroform for the preparation of CF_3_Cu, which participated in cross-coupling reactions with ArB(OH)_2_ in air [49]. Starting from CF_3_CO_2_Et or C_2_F_5_CO_2_Et, Mikami et al. obtained CF_3_Cu [44] or C_2_F_5_Cu [45]. The substrate scope of trifluoromethylation and pentafluoroethylation suggests that CF_3_Cu and C_2_F_5_Cu reagents are useful C*_n_*F_2_*_n_*_+1_^−^ sources for perfluoroalkylation reactions. Furthermore, CF_3_Cu and C_2_F_5_Cu were utilized for oxidative perfluoroalkylation reactions of arylboronic acids [44–45] (Scheme 13).

### Copper-catalyzed group transfer from fluoral derivatives

**Scheme 14:**
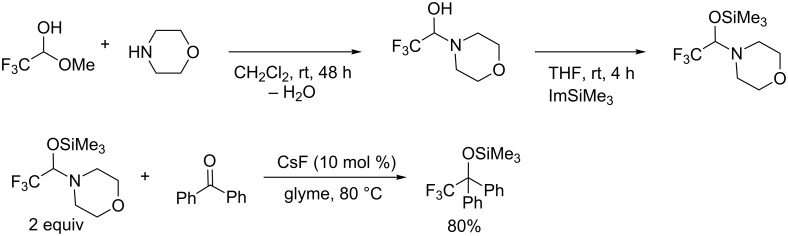
Trifluoromethylation with silylated hemiaminal of fluoral.

Catalytic systems in organic synthesis are desirable from an environmentally benign point of view. With regard to aromatic trifluoromethylation, the effort is devoted to reduce the copper reagents employed in the reactions. Copper-catalyzed aromatic trifluoromethylation with CF_3_SiMe_3_ was developed using phen as a ligand [50]. On the other hand, Billard and Langlois et al. described silylated hemiaminals of fluoral (trifluoroacetaldehyde) that act as a nucleophilic trifluoromethyl source for electrophiles such as aldehydes and ketones [51–52] (Scheme 14).

**Scheme 15:**
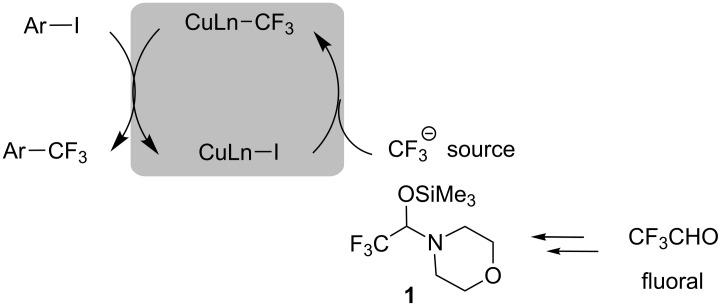
Catalytic trifluoromethylation with a fluoral derivative.

Amii and co-workers reported a copper-catalyzed aromatic trifluoromethylation from silylated hemiaminals of fluoral [53] (Scheme 15). Hemiaminal derivative **1** is readily prepared from commercially available CF_3_CH(OH)(OEt), which is a fluoral equivalent, and morpholine [52].

**Scheme 16:**
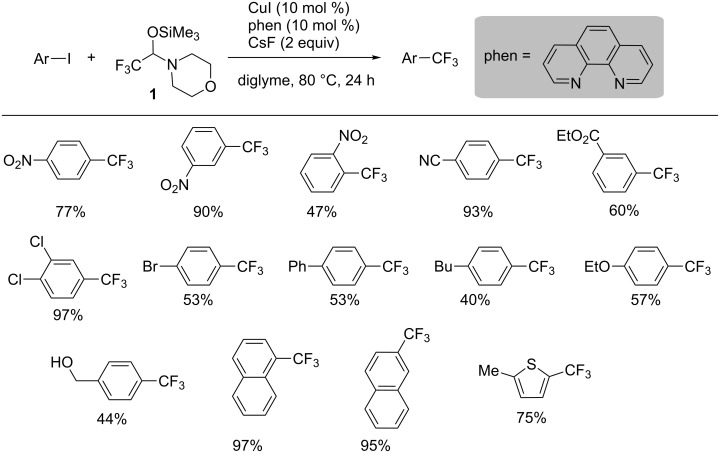
The scope of Cu-catalyzed aromatic trifluoromethylation. The yield was determined by ^19^F NMR analysis using (trifluoromethoxy)benzene as an internal standard.

The substrate scope of the catalytic trifluoromethylation is shown in Scheme 16. Nitro, cyano, and ester groups in iodoarenes were tolerable under the reaction conditions of copper-catalyzed nucleophilic trifluoromethylation. Electron-rich iodoarenes underwent the nucleophilic trifluoromethylation to afford the corresponding trifluoromethylated benzenes. Furthermore, the trifluoromethyl group was introduced into naphthalenes and thiophene with hemiaminal **1**.

**Scheme 17:**
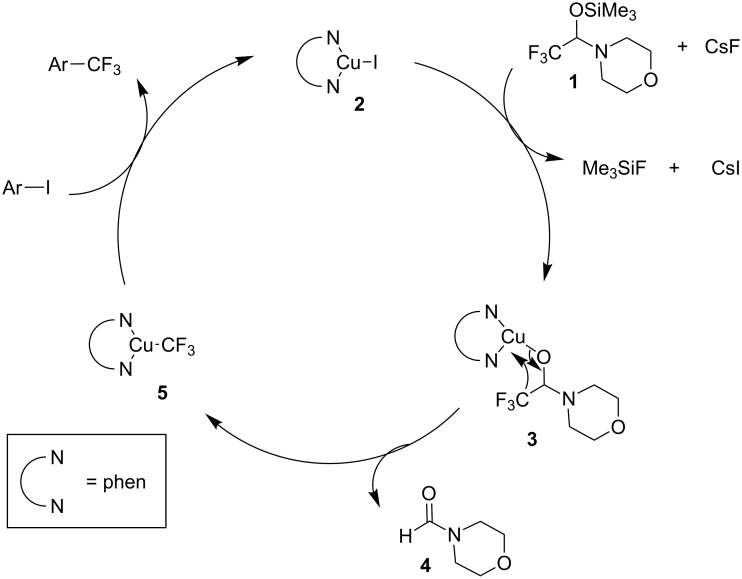
Plausible mechanism of Cu-catalyzed aromatic trifluoromethylation [53].

A catalytic amount of copper was enough to complete the reactions. In the synthesis of trifluoromethylarenes (Ar–CF_3_), the cross-coupling proceeded via the pathway shown in Scheme 17 [53]. First, the fluoride-ion-induced reaction of hemiaminal **1** with CuI-diamine complex **2** gave copper alkoxide **3**. Then the trifluoromethyl group in **3** migrates to generate the trifluoromethylcopper(I) complex **5** with the elimination of *N*-formylmorpholine (**4**) [54]. Finally, Ar–CF_3_ is formed by the coupling of CF_3_Cu complex **5** with Ar–I, and CuI-diamine complex **2** is regenerated.

## Conclusion

Fluorine has greatly contributed to the advancement of human life and the global demand for organofluorine compounds will continue to increase. Therefore, the introduction of fluorine-containing functional groups into organic molecules is recognized as a general strategy for the design of drugs and functional materials. In fact, the research activity on selective fluorination and trifluoromethylation has reached a mature state. The progress in fluoroalkylation of organic compounds could be accelerated by the use of fluoroalkylating reagents, which are inexpensive and easy to handle. Perfluoroalkyl carboxylic acid derivatives, such as perfluoroalkyl acetates, trifluoroacetic acid, chlorodifluoroacetates, trifluoromethyl ketones and hemiaminals of trifluoroacetaldehyde, are attractive perfluoroalkyl anion sources for aromatic perfluoroalkylation reactions. The generation of perfluoroalkylcopper from perfluoroalkyl carboxylic acid derivatives via carbon–carbon bond cleavage demands a high reaction temperature or basic conditions. Nevertheless, the simplicity of the operation and the reliability of higher yields would help the synthesis of fluorinated compounds in various fields.
